# Application of wings interferential patterns (WIPs) and deep learning (DL) to classify some *Culex*. spp (Culicidae) of medical or veterinary importance

**DOI:** 10.1038/s41598-025-08667-y

**Published:** 2025-07-01

**Authors:** Arnaud Cannet, Camille Simon Chane, Aymeric Histace, Mohammad Akhoundi, Olivier Romain, Pierre Jacob, Darian Sereno, Marc Souchaud, Philippe Bousses, Denis Sereno

**Affiliations:** 1 Direction Générale de la Santé, Paris, France; 2 ETIS UMR 8051, ENSEA, CNRS, CY Cergy Paris University, 95000 Cergy, France; 3 Parasitology-Mycology, Hopital Avicenne, AP-HP, Bobigny, France; 4 Cergy Paris University, Cergy, France; 5 CNRS, Bordeaux INP, LaBRI, UMR 5800, University of Bordeaux, 33400 Talence, France; 6 42, Paris, France; 7 MIVEGEC, CNRS, IRD, University of Montpellier, Montpellier, France; 8 UMR177 Intertryp, Institut de Recherche pour le Développement, CIRAD, University of Montpellier, Montpellier, France; 9 GoInsEct: Global Infectiology and Entomology Research Group, Montpellier, France

**Keywords:** Invasive species, Taxonomy, Entomology

## Abstract

In this paper, we test the possibility of using Wing Interference Patterns (WIPs) and deep learning (DL) for the identification of *Culex* mosquitoes species to evaluate the extent to which a generic method could be developed for surveying Dipteran insects of major importance to human health. Previous applications of WIPs and DL have successfully demonstrated their utility in identifying *Anopheles*, *Aedes*, sandflies, and tsetse flies, providing the rationale for extending this approach to *Culex*. Accurate identification of these mosquitoes is crucial for vector-borne disease control, yet traditional methods remain labor-intensive and are often hindered by cryptic species or damaged samples. To address these challenges, we applied WIPs, generated by thin-film interference on wing membranes, in combination with convolutional neural networks (CNNs) for species classification. Our results achieved over $95\%$ genus-level accuracy and up to $100\%$ species-level accuracy. Nonetheless, challenges with underrepresented species emphasize the need for larger datasets and complementary techniques such as molecular barcoding. This study highlights the potential of WIPs and DL to enhance mosquito identification and contribute to scalable tools for broader surveys of health-relevant Dipteran insects.

## Introduction

Pathogens transmitted by arthropods, including viruses, bacteria, and parasites, are among the most destructive infectious agents globally. Blood-feeding insects of the *Culex* genus (Linnaeus, 1758) are recognized vectors of significant diseases, such as West Nile virus fever, Japanese encephalitis, Saint Louis encephalitis, or lymphatic filariasis as examples (*Wuchereria bancrofti*)^1^. The genus encompasses 26 subgenera with over 783 recognized species and 55 subspecies, as recorded in the Integrated Taxonomic Information System (last accessed November 28, 2024, https://www.itis.gov). Many of these species transmit pathogens of medical or veterinary importance (see Table 1).

**Table 1:** Culex species of medical or veterinary interest.

Recognized vector competence	Reference
*Culex (Culex) antennatus*	https://wrbu.si.edu/vectorspecies/mosquitoes/antennatus
*Culex (Culex) fuscocephala*	https://wrbu.si.edu/vectorspecies/mosquitoes/fuscocephala
*Culex (Culex) gelidus*	https://www.wrbu.si.edu/vectorspecies/mosquitoes/gelidus
*Culex (Culex) nigripalpus*	https://www.wrbu.si.edu/vectorspecies/mosquitoes/nigripalpus
*Culex (Culex) perexiguus*	https://www.wrbu.si.edu/vectorspecies/mosquitoes/perexiguus
*Culex (Culex) pipiens*	https://wrbu.si.edu/index.php/vectorspecies/mosquitoes/pipiens
*Culex (Culex) quinquefasciatus*	https://wrbu.si.edu/index.php/vectorspecies/mosquitoes/quinquefasciatus
*Culex (Culex) restuans*	https://www.wrbu.si.edu/vectorspecies/mosquitoes/restuans
*Culex (Culex) salinarius*	https://www.wrbu.si.edu/vectorspecies/mosquitoes/salinarius
*Culex (Culex) sitiens*	https://www.wrbu.si.edu/vectorspecies/mosquitoes/sitiens
*Culex (Culex) tarsalis*	https://www.wrbu.si.edu/vectorspecies/mosquitoes/tarsalis
*Culex (Culex) theileri*	https://wrbu.si.edu/index.php/vectorspecies/mosquitoes/theileri
*Culex (Culex) tritaeniorhynchus*	https://wrbu.si.edu/index.php/vectorspecies/mosquitoes/tritaeniorhynchus
*Culex (Culex) univittatus*	https://wrbu.si.edu/index.php/vectorspecies/mosquitoes/univittatus
*Culex (Culex) vishnui*	https://www.wrbu.si.edu/vectorspecies/mosquitoes/vishnui
*Culex (Melanoconion) erraticus*	https://wrbu.si.edu/vectorspecies/mosquitoes/erraticus
*Culex (Melanoconion) ocossa*	https://wrbu.si.edu/vectorspecies/mosquitoes/ocossa
*Culex (Melanoconion) spissipes*	https://wrbu.si.edu/vectorspecies/mosquitoes/spissipes
*Culex (Melanoconion) taeniopus*	https://wrbu.si.edu/vectorspecies/mosquitoes/taeniopus
*Culex (Melanoconion) vomerifer*	https://wrbu.si.edu/vectorspecies/mosquitoes/vomerifer
*Culex (Oculeomyia) bitaeniorhynchus*	https://wrbu.si.edu/vectorspecies/mosquitoes/bitaeniorhynchus
*Culex (Oculeomyia) poicilipes*	https://wrbu.si.edu/index.php/vectorspecies/mosquitoes/poicilipes
**Data on vector competence**	
*Culex (Culex) annulus*	^2^
*Culex (Culex) australicus*	^3^
*Culex (Culex) coronator*	^4^
*Culex (Culex) decens*	^5^
*Culex (Culex) declarator*	^6^
*Culex (Culex) neavei*	^7^
*Culex (Culex) pseudovishnui*	^8^
*Culex (Culex) saltanensis*	^9^
*Culex (Culex) thriambus*	^10^
*Culex (Culex) torrentium*	^11^
*Culex (Culex) zombaensis*	^12^
*Culex (Lophoceraomyia) rubithoracis*	^13^
*Culex (Melanoconion) adamesi*	^14^
*Culex (Melanoconion) cedecei*	^15^
*Culex (Melanoconion) gnomatos*	^16^
*Culex (Melanoconion) panacossa*	^15^

The vectorial status of Culex species was collected from the Walter Reed Biosystematics Unit research site (last accessed on November 20/ 2024, https://wrbu.si.edu/), as well as from Google and PubMed (last accessed on November 20/2024)

They belong to four subgenera (*Culex*, *Melanoconion*, *Oculeomyia*, and *Eumelanomyia*), which include 22 species known to transmit pathogens of medical or veterinary significance. Key species, such as *Cx. quinquefasciatus* and *Cx. tritaeniorhynchus*, serve as vectors of West Nile virus, Japanese encephalitis virus, and other arboviruses. An additional 16 species are suspected vectors based on published data. Traditional morphological identification is labor-intensive and relies on diagnostic features and determination keys. This method is often challenged by cryptic species, overlapping morphological traits, and damaged specimens^17^. These limitations emphasize the need for innovative identification methods to enhance entomological surveys.

Insects with thin, transparent wing membranes—typically smaller species—can exhibit visible color patterns caused by thin-film interference. When these wings are illuminated in a dark, light-absorbing setting (such as under direct sunlight), striking interference patterns become visible across the membrane surface. These Wing Interference Patterns (WIPs) show substantial variation between different species, while remaining relatively consistent within a species or between sexes. The resulting coloration, reminiscent of the hues seen on soap bubbles, is linked to the local thickness of the wing membrane. Unlike conventional iridescence from flat films, which changes with viewing angle, the microstructure of insect wings functions as a dioptric system that stabilizes the interference pattern, making WIPs largely insensitive to viewing angle^18^. Because of their species-specific consistency and interspecific variability, WIPs hold strong potential as morphological markers for insect classification, offering a promising alternative or complement to traditional taxonomic traits.

Many research groups are actively developing computer vision and machine learning methods for automated insect identification, including techniques based on whole-body images^19^, geometric morphometrics^20^. These approaches have demonstrated notable success in identifying various taxa across different insect orders. Our work complements these broader efforts by focusing specifically on the use of Wing Interference Patterns (WIPs) as stable and species-specific markers. By integrating WIPs with CNN-based classification, we provide an alternative and robust framework for mosquito identification that could be particularly valuable for species with cryptic morphological traits or for damaged specimens. WIPs have been successfully applied to various insect groups, including *Glossina*, *Aedes*, *Anopheles*, *Phlebotomus*, and *Sergentomyia*^18,21–26^. Building on these successes, we investigated the use of WIPs and CNNs for the classification of *Culex* species, a critical genus for vector-borne disease control.

Our approach aims to test whether the combination of WIPs and deep learning can serve as a reliable and scalable method for identifying *Culex* species and explore to what extent this method could be generalized to survey a broader range of Dipteran insects of major relevance to human health.

## Results

### Training and classification

We evaluated the classifier using a dataset of *Culex* species, non-*Culex* Culicidae (Psychodidae, Glossinidae, and Ceratopogonidae) and other Culicidae (*Aedes*, *Anopheles*) as negative controls. The initial database comprised 572 images of 12 species across 5 subgenera. Only species with at least 10 images were retained, resulting in a refined dataset of 553 images representing WIPs from 7 species (Table 2) for training purposes.

**Table 2:** Culex included in the dataset.

Culex in our database	Med/vet int	Country code	Expert/year	Nb
*Culex (Culex) brumpti*	No	504	Bailly-Choumara/1965	2
Culex (Culex) decens**	Yes	854	J Hamon/1959	9
*Culex (Culex) quinquefaciatus*	Yes	250, 638	A Cannet, Ph Bousses/2012	68
*Culex (Culex) neavei*	Yes	638	Ph Bousses/2012	259
Culex (Culex) pipiens**	Yes	ND	ARIM	1
*Culex (Culex) thalassius*	No	24	G Legoff/2010	10
*Culex (Culex) tritaenhiorhynchus*	Yes	638	Ph Bousses/2012	166
*Culex (Culex) univitattus*	Yes	638, 450	Ph. Bousses/2012	22
Culex (Culiciomyia) nebulosus**	No	ND	ARIM	10
*Culex (Eumelanomyia) insignis*	No	638	Ph Bousses/2012	18
Culex (Maillotia) hortensis**	No	ND	ARIM	6
*Culex (Neoculex) territans*	No	ND	ARIM	1

** Samples not identified at the subspecies level. ARIM, sample deposited in the collection after identification. ARIM, specimen identified at the species level and deposited in the ARIM collection without further information

### Classification performance

The CNN achieved genus-level classification accuracy exceeding $95.00\%$ (Table 3).

**Table 3:** Test for accuracy of the DL (deep learning) process for the *Culex* (Linnaeus, 1758) genus identification.

		Predicted		
		*Culex* spp	Other	Nb of pictures
Truth	*Culex* spp	108 ($95.6\%$)	5	113
	Other	8	897 ($99.1\%$)	905

At the species level, performance varied, with perfect accuracy ($100.00\%$) for *Cx. neavei *and high accuracy ($75.00\%$ to $94.00\%$) for *Cx. insignis*, *Cx. quinquefasciatus*, and *Cx. tritaeniorhynchus*. Misclassification occurred for *Cx. thalassius* and (accuracy $0.00\%$), while low $40.00\%$ or moderate accuracy ($50.00\%$) were recorded for *Cx. univittatus* and *Cx. nebulosus* respectively 4.

**Table 4:** Test for accuracy of the DL (deep learning) process for the *Culex* (Linnaeus, 1758) species identification, in percentage of accuracy.

					Predicted					
	Species	ins	neav	neb	quinq	thal	trit	univ	Others	Nb
	ins	**75.0**	0.0	0.0	0.0	0.0	0.0	0.0	25.0	4
T	neav	0.0	**100.0**	0.0	0.0	0.0	0.0	0.0	0.0	52
r	neb	0.0	0.0	**50.0**	0.0	0.0	0.0	0.0	50.0	2
u	quinq	0.0	7.1	0.0	**92.9**	0.0	0.0	0.0	0.0	14
t	thal	0.0	0.0	0.0	0.0	**0.0**	0.0	0.0	100.0	2
h	trit	0.0	0.0	0.0	2.9	0.0	**94.1**	0.0	2.9	34
	Univ	0.0	40.0	0.0	0.0	0.0	20.0	**40.0**	0.0	5
	Others	0.0	0.6	0.1	0.1	0.0	0.1	0.0	**99.1**	905

*Cx. insignis*; neav, *Cx. neavei*; neb, *Cx. nebulosus*; quinq, *Cx. quinquefaciatus*; thal, *Cx. thalassius*; trit, *Cx tritaenorhynchus*; univ, *Cx. univittatus*; Others, other culicidae species not belonging to the *Culex* genus; Nb, number of pictures tested.

In a multi-country external quality assessment conducted within the MediLabSecure Network, morphological identification of mosquitoes achieved an average species-level accuracy of $64\%$, with considerable variability observed across participating laboratories^27^. Our CNN-based method achieved species-level accuracy ranging from 40 to 100%, depending on the *Culex* species. While these results can surpass the performance of morphological identification reported in that assessment, accuracy varies depending on the dataset, methodological approach, and study context. Considering this baseline, we interpret species identification accuracy above $65\%$ as indicative of good performance, as it exceeds the mean accuracy achieved by trained entomologists in the referenced assessment. Notably, while our model’s performance in recognizing samples belonging to the *Culex* genus was variable, it remained above $65\%$ for 4 species, and below $<65\%$ for 3 species tested. For these latter species, the accuracy achieved by our model was nonetheless lower than that reported in other AI/ML-based studies^28^.

## Discussion

This study demonstrates the potential of integrating Wing Interference Patterns (WIPs) with deep learning models for classifying *Culex* species. By combining the distinct, species-specific patterns of WIPs with the analytical capabilities of CNNs, we achieved high genus-level classification accuracy and variable species-level performance.

Species-level accuracy varied due to dataset limitations, particularly for poorly represented species like *Cx. thalassius* and *Cx. univittatus* or *Cx nebulosus*. Expanding the dataset to include more specimens and diverse conditions–such as age, preservation state, and environmental origin–could improve accuracy. Integrating complementary techniques like molecular barcoding^29^ or protein profiling^30^ can enhance dataset robustness and address cryptic species identification.

This approach holds significant promise for improving vector surveillance. Accurate identification of key vector species, such as *Cx. quinquefasciatus* and *Cx. tritaeniorhynchus*, supports efforts to monitor and control vector-borne diseases. The scalable and cost-effective nature of WIP imaging makes it suitable for large-scale biodiversity monitoring and entomological surveys. By standardizing imaging protocols and providing user-friendly tools for field researchers, this method can become a practical asset for global health initiatives.

Several areas require further exploration to enhance the reliability of this approach on *Culex*, including dataset expansion, integration of molecular techniques, and standardization of imaging. Expanding the dataset by increasing species representation and capturing greater variability in specimen conditions is crucial for improving classification accuracy. Incorporating molecular techniques, such as DNA barcoding and proteomic profiling, can help resolve cryptic species and provide additional discriminatory features to complement Wing Interference Patterns (WIPs). Additionally, standardizing imaging protocols is essential to minimize variability and ensure consistent image quality, which will ultimately enhance the performance and robustness of deep learning models. Another critical consideration for future research is the establishment of standardized criteria for evaluating the accuracy of AI/ML-based mosquito identification systems. Currently, differences in dataset composition, feature sets, and methodological approaches can hinder direct comparisons across studies. Defining consistent evaluation frameworks, such as accuracy thresholds, dataset curation guidelines, and performance metrics, will facilitate reliable benchmarking and enhance the reproducibility of AI/ML applications in entomological surveillance. By harmonizing these criteria, future studies can provide clearer insights into the true strengths and limitations of methods, to drive progress in mosquito vector identification.

The misclassification of *Culex. nebulosus*, *Cx. thalassius* and *Cx. univittatus* underscores the need to reassess diagnostic criteria and expand molecular datasets for these species. Such efforts would improve model robustness and generalizability, enabling broader applications across taxa and geographic regions.

This study demonstrates the effectiveness of combining Wing Interference Patterns (WIPs) with deep learning models for identifying important *Culex* vectors. High genus-level accuracy and reliable species-level results demonstrate the effectiveness of WIP-based classification for taxonomic applications, highlighting its potential as a scalable and cost-effective tool for *Culex* vector surveillance. By enhancing species identification capabilities, this method can significantly contribute to global health efforts in mitigating vector-borne diseases.

Furthermore, the combination of WIPs and deep learning-based identification presents a promising avenue for enhancing large-scale entomological surveys of Dipteran insects with medical or veterinary relevance. Its minimally invasive and high-throughput potential could offer an innovative tool for biodiversity monitoring, supporting more efficient species identification at broader scales.

To fully realize this potential, the system must now be rigorously challenged by incorporating additional family member species, aiming to include the highest biodiversity possible across Dipteran insects. Expanding datasets to encompass greater taxonomic diversity and ecological variability will not only strengthen model robustness but also ensure its applicability across a wide range of species and geographic contexts.

Future research should also integrate complementary approaches, such as molecular barcoding and proteomic profiling, to resolve challenges posed by cryptic species and underrepresented taxa. Refining imaging protocols and classification algorithms will further enhance the accuracy and generalizability of this method.

By bridging cutting-edge machine learning with innovative imaging techniques, the WIPs and DL method has the potential to become a transformative tool for vector surveillance and biodiversity research, advancing global health and ecological conservation efforts.

## Methods

### *Culex* collection and storage

The reference collection of *Culex* for this study includes samples from the ARIM collection (https://arim.ird.fr/) maintained by the Institut de Recherche pour le Développement (IRD). Furthermore, field-collected specimens, initially identified through regional morphological keys at the time of capture and included in the ARIM collection, were also incorporated into the database. A detailed description of these samples is provided in Table 2.

### Image acquisition and database construction

The same standard operating procedures (SOP) previously employed for capturing WIPs of *Glossina*, *Anopheles*, *Aedes* and *Psychodidae* were applied to Culex as described in reference^31^. The procedure involved dissecting the wings and mounting them on glass slides. A coverslip was applied, and the specimens were photographed using an xVH-Z20r camera with a VH K20 adapter ($\hbox {Keyence}^{TM}$) set to a $10^{\circ }$ illumination angle. The High Dynamic Range (HDR) function was utilized for all images. Each photograph was cropped to standardize the size and exclude wing dimensions as a factor in species identification for deep learning applications. Metadata, including geographical origin, collection date, species identity, specimen sex, and the entomologist’s name who identified the sample, were recorded. The camera settings were: white balance 3200 K, shutter speed 1/15 sec, gain 0db, frame rate 15F/s, brightness $15\%$, texture $15\%$, contrast $45\%$, and color $100\%$. Adjustments for luminosity, contrast, shadows, reflection, and saturation were set to $80\%$, $100\%$, $0\%$, $0\%$, and $100\%$, respectively, using Windows 7 Home Edition. Before finalizing the images in the database, dust removal was performed manually.

### Collected dataset, image pre-processing, and dataset splitting for training/learning and validation

An annotated image dataset was created, comprising 572 images representing 12 species, for use in *Culex* classification. For model generalization purposes, variables such as sex, geographic origin, age, and physiological state (whether blood-fed or not) were excluded from consideration. Specimens genus was not considered to build the training and test datasets. In addition, 4,944 images of WIPs from various Diptera families, excluding *Culex* members, were incorporated. Species with fewer than ten images were omitted from species-level training to avoid overfitting . All images were resized to 256 x 116 pixels, and pixel values were normalized within the range of (0,1) as previously published^31^.

**Figure 1:**
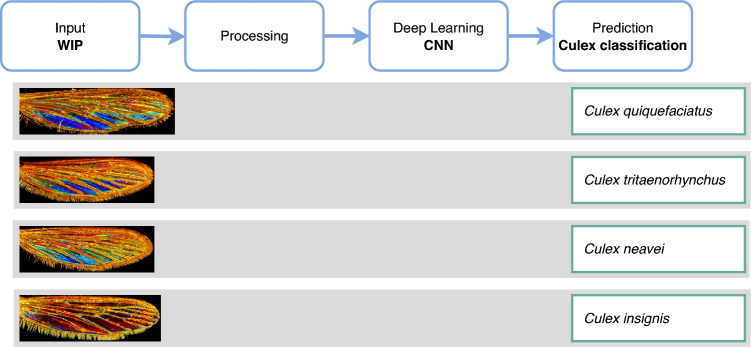
Upper panel: schematic representation of the classification pipeline; lower panel: WIP images of selected *Culex* species. The pipeline illustrates the Convolutional Neural Network (CNN)-based approach used for species identification. The classification process involves two main steps: extraction of hierarchical features via convolutional layers, followed by classification using fully connected and softmax layers. Feature maps are visualized by weighting them with channel-wise averaged gradients.

A range of image augmentation methods was employed, including vertical and horizontal flips, random rotations, and zoom transformations. The primary goal of these techniques was to enhance the robustness of the WIPs dataset by introducing randomized variations, thereby increasing the diversity of training examples without collecting new data. The dataset underwent k-fold cross-validation (k=5), a widely used method to assess the robustness of machine learning techniques, including deep learning. This process was conducted similarly to the previous analyses of Glossina WIPs in reference^32^ and illustrated in the Fig. 1. The dataset was randomly shuffled and divided into k equal-sized subsets with similar class distributions. For each fold, a classifier was trained on k-1 subsets and validated using the remaining kth subset, allowing for the calculation of the mean accuracy across the five generated classifiers.

Among all machine learning approaches, Deep Convolutional Neural Networks (CNNs) and their architectural variations have proven to be the most effective for image classification in recent years. Unlike traditional shallow methods (such as Support Vector Machines, Random Forests, and Boosting-based approaches), CNNs do not require hand-crafted features as input. Instead, the method automatically selects the optimal features during the learning process, making it particularly suitable for WIP classification tasks.

### Training of the convolutional neural network (CNN)

The original CNN architectures, including MobileNet, ResNet, and YOLOv2, were selected for automatic classification using the dataset described above. In contrast to conventional deep learning models, our approach is more compact, designed specifically to handle the smaller size of our dataset. To address this, we developed streamlined image recognition and classification architectures. The first architecture draws inspiration from MobileNet, leveraging depth-wise convolution^33^. Our model employs only a single depth-wise convolution per layer to reduce complexity and the number of extracted features. Batch normalization was applied to expedite and stabilize the training process^34^. This compact CNN architecture, based on MobileNet, incorporates two interconnected layers similar to VGG40, as used in YOLOv2, with a DarkNet-1938 architecture. Since such deep architectures often tend to overfit the training data (limiting generalization to new datasets), we tested two simplified architectures with fewer scales than the original. These were designated DarkNet-9 (with 8 convolution layers and one classification layer) and DarkNet-14 (with 13 convolution layers and one classification layer). Additionally, we replicated the ResNet18 architecture37, initializing it from scratch. Despite its depth, which may increase the risk of overfitting, the residual connections in ResNet^35^ facilitated successful convergence of the training process.

We also implemented a traditional approach based on extracting SURF descriptors (an efficient variant of SIFT descriptors), employing a Bag of Features (BoF) representation with a dictionary of 4000 codewords, and using an SVM with a polynomial kernel. For each task, we utilized a single fully connected layer with softmax activation to predict the probability of an image belonging to the correct class. The networks were trained using Stochastic Gradient Descent (SGD) with a learning rate of 10e2 and a momentum of 0.9 for 30 epochs. The method was implemented on a workstation equipped with a quad-core CPU at 3.0 GHz and 16 GB of RAM. Further details on training options, accuracy, sensitivity, and the code can be found at (https://github.com/marcensea/diptera-wips/commit/12f39ab500a3f820cfb817c67ef25c580942301d). Combining the dataset repositories^36,37^ allows for the collection of 5,516 pictures from 7 families (Culicidae, Calliphoridae, Muscidae, Glossinidae, Tabanidae, Ceratopogonidae, and Psychodidae) and 21 genera, from which the analysis of *Culex* was performed.

## Data Availability

Combining the dataset repositories (https://doi.org/10.6084/m9.figshare.24444937.v2) and (https://doi.org/10.6084/m9.figshare.22083050.v4) allows for the collection of 5,516 pictures from 7 families (Culicidae, Calliphoridae, Muscidae, Glossinidae, Tabanidae, Ceratopogonidae, and Psychodidae) and 21 genera, from which the analysis of Culex was performed. Accession codes: The source code is publicly available on GitHub, with a direct https://github.com/marcensea/diptera-wips.git.
